# An important ‘step’ towards the standardisation of care offered by physiotherapists working in an ICU in a low- to middle-income country

**DOI:** 10.7196/AJTCCM.2021.v27i3.159

**Published:** 2021-10-04

**Authors:** I S Kalla

**Affiliations:** Department of Internal Medicine, School of Clinical Medicine, University of the Witwatersrand, Johannesburg, South Africa

## Editorial

The discipline of critical care medicine has evolved from being a Cinderella speciality to one offering support to our most sick and vulnerable patients. These patients are referred from the various departments of medicine and the critical care physician/intensivist must attend to them, irrespective of which department has referred them into the intensive care unit (ICU). This evolution of critical care medicine as a stand-alone division has been accompanied by the establishment of unique management protocols and the use of several new (or repurposed) lifesaving equipment to allow the intensivist to master his trade in saving lives. However, the intensivist functions predominantly as the ‘captain’ of the ICU and is reliant on all supporting staff to aid him in managing the ICU patient. There is a constant flux in ICU management protocols and the introduction of antibiotic stewardship and infection control measures has brought critical care to the cutting edge of medicine. This is further complicated by the use of novel mechanical devices for various forms of organ support such as ventilators, continuous renal replacement/ dialysis machines and extracorporeal membrane oxygenators. Hence, the ICU environment can appear very intimidating, if not hostile, to the unfamiliar.^[1]^

The training of intensivists and critical care nurses has evolved with an increasing understanding of the discipline of critical care medicine. This has led to the establishment of set curricula and regulatory boards with exit exams maintaining a standard of competency.^[2]^ However, within the ICU environment, the ancillary supporting staff including physiotherapists, occupational therapists, dieticians and pharmacists form an integral part of the multidisciplinary team.^[3]^ Unfortunately, they are often neglected in the ongoing continuous professional development programmes within the ICU environment. Physiotherapy is one of the most important non-pharmacological supportive therapies administered to patients in the ICU.^[4]^The major goal of physiotherapy in the ICU includes maintaining/restoring the general patient’s functional capacity and restoring respiratory and physical independence.^[5–7]^

In Nigeria, there are no specific guidelines governing physiotherapy practice in ICUs. In this issue of the AJTCCM, Idris *et al*.^[8]^ have published the outcome of a Delphi survey attempting to address this lack of a critical care curriculum for physiotherapists.^[9]^ A previously validated questionnaire comprising 222 question items on the role of physiotherapy in critical care developed by Skinner *et al*.^[10]^ was adopted and administered to participants over three rounds via an online Delphi survey.^[11]^ To ensure validity of the responses, the eligibility of participation was limited to physiotherapists with a minimum of 5 years’ working experience, 3 of which must have been in a senior role within the critical care setting, or physiotherapists involved in the supervision or teaching of physiotherapy staff working on call or completing emergency duty. Furthermore, the participants were restricted to academic physiotherapy staff involved in the provision of entry-level cardio-respiratory physiotherapy, with at least two articles published in critical care. Twenty-six experts consented and completed the first round of the study answering either ‘essential’, ‘not essential’ or ‘unsure’ for each question item. Interestingly, of the 222 questions circulated, 178 question items were adjudged to be essential but none of the question items were ranked as ‘not essential’ after all the rounds. For each question item, a consensus to determine if such a question item was either essential or not essential was based on a consensus agreement of 70%.^[12]^ The consensus derived from this well-designed and previously validated questionnaire will form the framework for the development of a document/curriculum outlining the minimum clinical standard of practice for physiotherapists working in ICUs in Nigeria.

Idris *et al*.^[8]^ should be commended on this well-written publication. The noteworthy points from this publication are that the practice of critical care medicine needs to be standardised across all disciplines working within the ICU environment. The advent of Google forms distributed via social media platforms such as WhatsApp, Facebook and Gmail have revolutionised how consensus documents can be developed from a panel of experts in the field of practice. Finally, tools developed and validated in high-income countries can be equally effective when used in low- to middle-income countries, as the challenges faced with standardisation of care in the ICU are universal.^[13]^ This publication has set the framework and established a guideline for further ancillary support staff working with the ICU environment to adopt and use to improve the level of care offered to the most sick and vulnerable patients.^[14,15]^
